# Supplementary material for: Combination of Whole-Body Baseline CT Radiomics and Clinical Parameters to Predict Response and Survival in a Stage-IV Melanoma Cohort Undergoing Immunotherapy
Source: Cancers (Basel). 2022 Jun 17;14(12):2992. doi: 10.3390/cancers14122992 (PMC9221470; doi:10.3390/cancers14122992)
Supplement: Supplementary file 1 [file cancers-14-02992-s001.zip › cancers-1769106-supplementary.pdf]

cancers

MDPI

Table S1. CT scanner vendors.

	Scanner	Vendor	Number of patients
Cohort			
inhouse	SOMATOM Definition AS+	Siemens	56
	SOMATOM Definition Flash	Siemens	4
	SOMATOM Force	Siemens	92
	Sensation 64	Siemens	29
	Biograph 128	Siemens	38
external	Aquillion One	Canon	3
	Astelion	Canon	2
	Discovery 710	General Electrics	1
	LightSpeed VCT	General Electrics	1
	Optima CT540	General Electrics	1
	Optima CT660	General Electrics	1
	Ingenuity Core	Philips	1
	Biograph 128	Siemens	1
	Biograph 64	Siemens	4
	Emotion 16	Siemens	2
	Emotion 6	Siemens	2
	Perspective	Siemens	1
	Scope	Siemens	1
	Sensation 64	Siemens	3
	Sensation Cardiac	Siemens	1
	SOMATOM Definition AS	Siemens	15
	SOMATOM Definition Edge	Siemens	1
	SOMATOM Definition Flash	Siemens	1
	SOMATOM Force	Siemens	1
Total			262

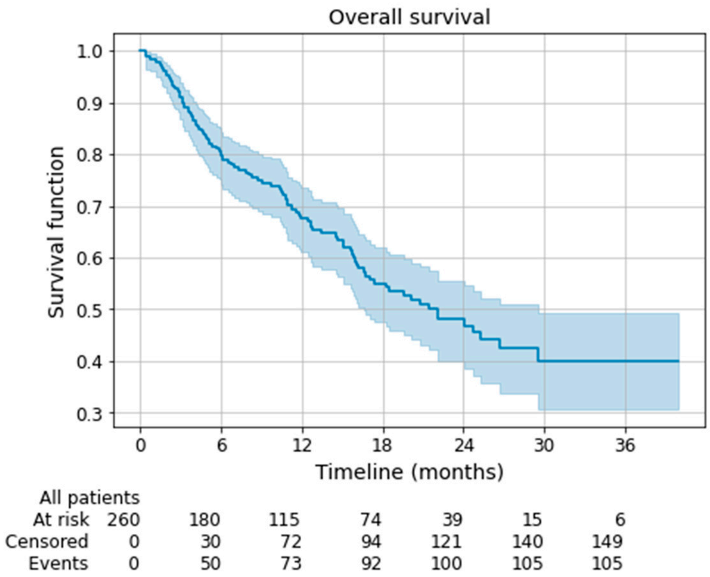

Figure S1. Kaplan-Meier estimator of overall survival for the whole cohort.
